# Effect of Orthostatic Tremor on Quality of Life – a Cohort Study

**DOI:** 10.5334/tohm.1008

**Published:** 2025-05-07

**Authors:** Wietske A. Babeliowsky, Bart Swinnen, Jeroen Hoogland, Rob M. A. de Bie, Anne-Fleur van Rootselaar

**Affiliations:** 1 Department of Clinical Neurophysiology, Amsterdam UMC, University of Amsterdam, Amsterdam, The Netherlands; 2 Department of Neurology, Amsterdam UMC, University of Amsterdam, Amsterdam, The Netherlands; 3 Department of Neurology, University Hospitals Leuven, Leuven, BE; 4 Department Epidemiology and Data Science, Amsterdam UMC, University of Amsterdam, Amsterdam, The Netherlands; 5 Amsterdam Neuroscience, Neurodegeneration, Amsterdam, The Netherlands

**Keywords:** Orthostatic Tremor, Quality of Life, Daily Functioning, Well-being, Long-term follow-up

## Abstract

**Background:**

Orthostatic tremor (OT) is characterized by a lower extremity tremor causing unsteadiness while standing, urging patients to lean, walk, or sit to ease symptoms. This severely disrupts daily life and reduces quality of life (QoL), though the extent of QoL impairment remains largely unknown. The objective of this cohort study was to evaluate the effect of OT on daily functioning and well-being over time.

**Methods:**

In this prospective single-center and community based cohort study, OT patients annually completed self-report scales from 2018 to 2024, including the Dutch OT-questionnaire, Hospital Anxiety and Depression Scale (HADS), (instrumental) Activities of Daily Living, and the Short Form-36 (SF-36) to evaluate QoL.

**Results:**

Fifty-three OT patients participated in the study. OT patients showed reduced well-being compared to the general population based on outcomes from the SF-36 and HADS, with 30 out of 53 patients experiencing potential or suspected depression and/or anxiety. Despite viewing OT as progressive, most patients saw no significant changes in overall well-being or daily functioning, although a significant difference was found between baseline and last follow-up for physical role limitation.

**Discussion:**

Although OT patients report reduced well-being, both daily functioning and overall well-being remained stable over time, despite progressive symptoms. This is likely due to patients increased ability to adapt to OT symptoms. Additionally, a substantial portion had potential or suspected depression or anxiety.

**Highlights:**

Orthostatic tremor (OT) patients report reduced quality of life, with worsened walking ability and increased weather sensitivity over time. Despite this, daily functioning and overall well-being remained stable throughout the study. A significant portion of patients also showed potential or suspected depression and/or anxiety.

## Introduction

Orthostatic tremor (OT) is a rare neurological disorder characterized by a lower extremity tremor of ≥13 Hz when standing [1,2]. OT is categorized as ‘primary OT’ when idiopathic, ‘secondary OT’ when linked to another disorder, or ‘OT plus’ when associated with other movement disorders [3]. Primary OT typically affects middle-aged and elderly individuals, has a female predominance and follows a progressive course [4,5,6]. The diagnostic criteria include: 1) a subjective sensation of unsteadiness while standing, 2) minimal clinical findings, and 3) surface electromyography (EMG) of the leg muscles, identifying the pathognomonic rhythmic bursts of ≥13 Hz during stance that revolves when sitting [1,2,7,8,9]. The high-frequency tremor causes unsteadiness while standing, leading to a fear of falling and prompting patients to lean on objects, walk, or sit to alleviate symptoms [3,10,11].

The inability to stand still can significantly affect patients’ lives, limiting daily activities [10,12]. The need to sit or move may cause patients to avoid situations requiring prolonged standing, like shopping or queuing [3]. There is no cure, and current treatments, including medications, deep brain stimulation (DBS), and spinal cord stimulation, offer limited relief and often cause troublesome adverse effects, further reducing quality of life (QoL) [5,6,13,14,15,16,17,18,19,20].

While it is clear that OT significantly affect patients’ lives, the precise extent of QoL impairment remains largely unexplored. To date, only three studies have assessed QoL in primary OT patients [12,21,22]. Two studies reported a severe compromise in QoL, with fear of falling identified as the primary predictor, however longitudinal data was lacking [12,21]. One study developed a novel tool to assess the impact of OT and evaluated long-term clinical progression, revealing a wide range of effects on patients’ QoL [22].

In this study, we conducted a comprehensive longitudinal analysis of a cohort of OT patients in the Netherlands, to evaluate the effect of OT on daily functioning and well-being over time using several validated measures. The findings aim to enhance understanding of this debilitating disorder, provide better patient educations, identify specific needs, and offer targeted support.

## Methods

### Patients

In this prospective cohort study patients were selected from our online registry, which includes individuals from the Netherlands OT Patient Support Group and Amsterdam University Medical Centers (Amsterdam UMC). We included patients who: had an EMG-confirmed diagnosis of primary OT with a known tremor frequency [1], completed the set of scales (within four months) at least once between February 2018 and August 2024, and provided written informed consent. Patients were excluded if they had potential secondary OT or OT plus, or if they had previously undergone DBS surgery, because this group has already been extensively reported [23].

Clinical characteristics, including sex, age at onset, diagnostic delay, and tremor frequency, were extracted from self-report scales and electronic patient files (EPF).

### Standard Protocol Approvals, Registrations, and Patient Consents

All patients provided written consent for their data to be used for this study, although the Institutional Review Board (IRB) of Amsterdam UMC waived the need for specific consent for scientific use of this data.

### Outcome measures and data collection

The following self-report scales were used:

– the Dutch OT-questionnaire (Supplementary File 1) which was developed in collaboration with the Netherlands OT Patient Support Group in 2014 and hosted on their website [24]. This 33-item questionnaire covers treatment, symptoms, and QoL, and was used in this study to assess symptom severity, the effect of OT on daily life and to identify factors influencing OT;
– Hospital Anxiety and Depression Scale (HADS) for evaluating anxiety and depression [25];
– Activities of Daily Living (ADL) and Instrumental Activities of Daily Living (iADL) scales for assessing disability [26,27,28];
– and Short Form-36 (SF-36) for measuring health-related QoL [29].

For patients receiving active clinical care at the OT center of expertise of Amsterdam UMC, scales were administered annually as part of routine clinical care, with additional data extracted from EPF. For patients not under active care at Amsterdam UMC, questionnaires were distributed annually during a meeting of the Netherlands OT Patient Support Group.

The first completion of the scales served as the baseline. Follow-up data were collected during clinical assessments at follow-up (FU) 1 (0–20 months after baseline), FU2 (>20–40 months after baseline), and FU3 (>40–60 months after baseline). This report includes data from baseline and follow-up assessments.

### Data analysis

The following outcome measures were included:

– Effect on daily functioning based on: 1) questions regarding impact of OT (Visual Analogue Scale, VAS), daily functioning and influencing factors (yes/no questions) from the OT-questionnaire (see Supplementary file 1 for details on specific questions used, and Table 2), 2) iADL and ADL, and 3) SF-36 domains ‘physical functioning’, ‘physical role limitation’, and ‘social functioning’;
– Effect on patient well-being based on: 1) the HADS, and 2) SF-36 domains ‘emotional role limitation’, ‘energy/fatigue’, ‘emotional well-being’, ‘pain’, ‘general health’, and ‘health change’.

For all analyses, duplicate sets (two or more sets from a single patient per FU-period) were removed, only including the most complete and appropriately timed set was selected. Missing values were not replaced in descriptive analyses. Since follow-up was inconsistent for some patients, group composition varied at each follow-up, and patient characteristics were noted at each time point and compared. SF-36 and HADS scores were compared to normative values. In addition, linear mixed models (LMM) and generalized linear mixed models (GLMM) were used to analyze the outcome measures over time.

The models contained a categorical fixed effect of time (FU with respect to baseline) and random intercepts at the patient level, and were adjusted for baseline age, gender, and disease duration. All analyses were conducted using R version 4.4.2.

## Results

### Patients

**Figure 1:**
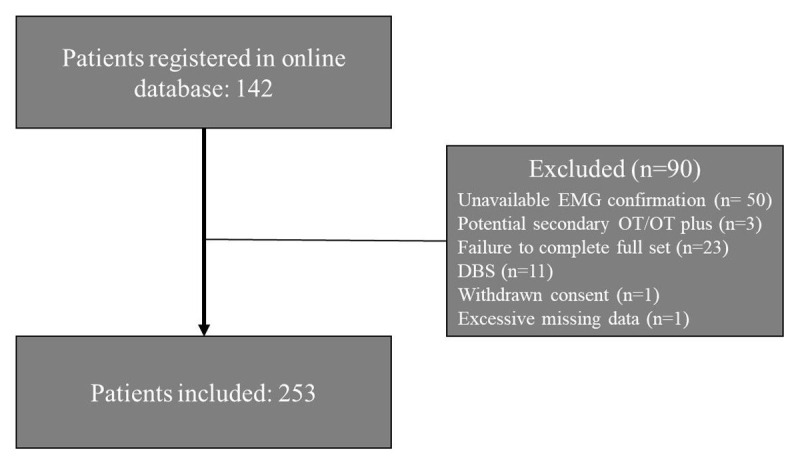
**Flow-chart patient inclusion**. Number of patients included, and reasons for exclusion.

Of the 142 patients registered in our online database, 53 were included in the study. Reasons for exclusion included: unavailable EMG confirmation (n = 50), potential secondary OT or OT plus (n = 3), failure to complete the full set of scales within four months (n = 23), DBS treatment (n = 11), withdrawal of consent (n = 1), and excessive missing data (n = 1) (Figure 1). Patient characteristics are presented in Table 1, with a significant difference between baseline and FU2 (t(72.25) = –2.13, p = .037, 95% CI of –8.65, –0.28), and baseline and FU3 (t(14.38) = –2.38, p = .032, 95% CI of –14.22, –0.76) for disease duration. The mean (±SD) age at inclusion was 67.5 ± 8.4 years and the majority of patients were female (31 out of 53, 58.5%).

**Table 1:** Patient characteristics.


CHARACTERISTICS	BASELINE	n	FOLLOW-UP 1	n	FOLLOW-UP 2	n	FOLLOW-UP 3	n

Amount of females (n)	31 (58.5%)	53	21 (56.8%)	36	20 (64.5%)	31	6 (60.0%)	10

Age at diagnosis (y)	64.0 ± 9.1	53	63.9 ± 9.3	36	64.4 ± 9.2	31	66.7 ± 8.4	10

Age at onset (y)	56.9 ± 11.3	52	56.9 ± 10.9	36	55.8 ± 11.7	30	59.8 ± 14.6	10

Diagnostic delay (y)	8.8 ± 7.2	52	7.8 ± 7.0	36	8.4 ± 6.7	30	10.2 ± 6.1	10

Mean age (y)*	67.5 ± 8.4	53	69.5 ± 8.5	36	71.3 ± 8.7	31	75.3 ± 5.0	10

Disease duration (y)**	2.6 ± 2.6	53	5.5 ± 3.7	36	7.0 ± 3.7	31	8.4 ± 4.6	10


Values are given either given in absolute numbers (percentage of total) or in mean ±SD. Abbreviations: n= number of patients. *= Average age at completion of questionnaires. **= Average disease duration at completion of questionnaires.

The mean follow-up duration was 28.0 months (range 8.8–59.3). Patients completed a mean of three sets of scales (range 1–7), with 44 patients having at least one follow-up assessment. From three patients a duplicate set was removed before analysis. FU1 occurred at a mean of 12.8 months (range 7.5–19.3), FU2 at 29.4 months (range 20.3–37.3), and FU3 at 48.3 months (range 46.3–59.3).

### Daily functioning

At baseline, most participants reported being mobile and engaged in physical activities. Specifically, 75% (36 out of 48 patients) reported a satisfactory walking ability, and 62% (26 out of 42 patients) were actively involved in sports and/or physical activities. Patients identified various factors influencing their OT symptoms, including fatigue (80%, 39 out of 49 patients), stress (71%, 35 out of 49 patients), and weather (25%, 12 out of 48 patients). Additionally, 86% (42 out of 49 patients) considered OT to be progressive (Table 2).

**Table 2:** Daily functioning over time.


OUTCOME MEASURE	BASELINE	n	FOLLOW-UP 1	n	FOLLOW-UP 2	n	FOLLOW-UP 3	n

Satisfactory walking ability	75%	48	67.5%	37	55.6%	29	57.1%	7

Sport(ing) activities	61.9%	44	48.7%	36	65.4%	29	71.4%	7

Influenced by fatigue	79.6%	49	90.0%	37	61.5%	29	71.4%	7

Influenced by stress	71.4%	49	70.0%	37	57.7%	29	85.7%	7

Influenced by weather	25%	48	25.6%	36	37.5%	27	42.6%	7

OT progressive	85.7%	49	86.1%	33	80%	28	85.7%	7

VAS score symptoms	6.4 (±2.4)	52	6.7 (±2.2)	37	6.3 (±2.1)	31	6.5 (±1.7)	10

VAS score impact	6.1 (±2.6)	51	6.7 (±2.4)	37	6.1 (±2.2)	30	6.6 (±1.9)	10

iADL	1.8 (±2.4)	52	2.1 (±2.7)	37	2.0 (±2.8)	31	1.8 (±2.4)	10

ADL	5.9 (±0.4)	53	6.0 (±0.2)	34	5.8 (±0.5)	30	6.0 (±0)	10


Values are given either in percentages based on the number of patients included in the analysis or in mean ± SD. Abbreviations: n = number of patients.

**Figure 2:**
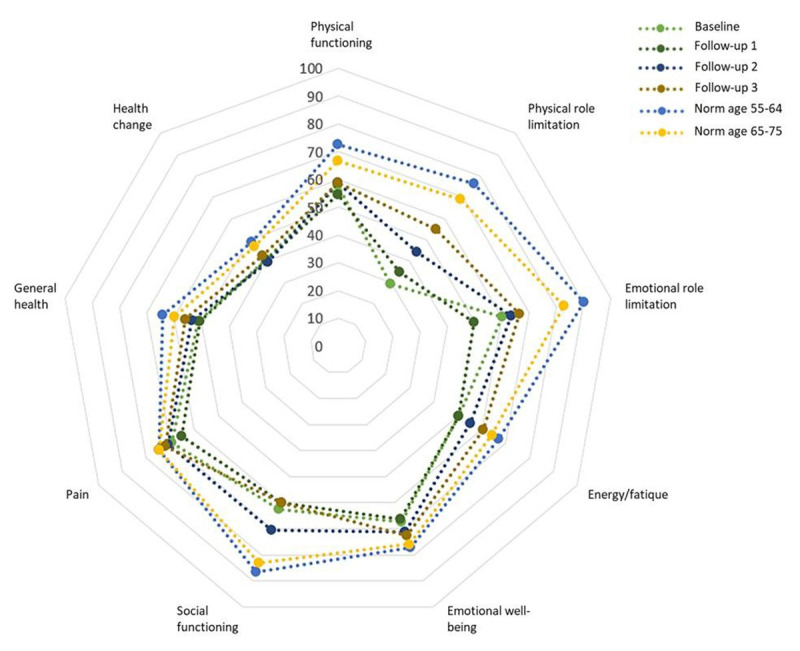
**SF-36 scores over time**. Baseline SF-36 scores were below age-related normal values. Upon follow-up, scores remained stable, with no to minimal differences over time, except for a significant difference between baseline and FU3 for ‘physical role limitation’. Abbreviations; FU = follow-up, SF-36 = Short Form 36.

The baseline SF-36 analysis revealed that the sub-scores for ‘physical role limitation’ and ‘social functioning’ were below age-related normal values (Figure 2).

During follow-up, descriptive statistics indicated a tendency for walking mobility to decline (57.1% at FU3 vs. 75.0% at baseline), and a trend towards more patients reporting their symptoms being influenced by the weather (42.6% at FU3 vs. 25.0% at baseline) (Table 2). However, the results of the GLMM showed no significant changes over time (Supplementary Table 1). For the SF-36, result of the LMM showed a significant effect of time (p = 0.04, df = 3, chi2 = 8.30), with scores increasing over time (significant difference between baseline and FU3 (55.0 at FU3 vs. 29.3 at baseline, p < 0.01205, and 95% CI of 6.45–48.22) for the sub-score ‘physical role limitation’ (Figure 2). Additionally, gender had a significant effect, with women scoring higher on ‘physical role limitation’ (p < 0.005). Substantial (unexplained) between-subject variability remained after adjustment for baseline age, gender, and disease duration. This variability is captured by the random intercepts in the mixed models (see Supplementary Table 1), and indicates that mixed models were indeed needed.

Fluctuations in scores over time were observed between subjects and across time points for sport participation, fatigue, stress as factors influencing OT symptoms, and the SF-36 sub-score ‘social functioning’ but no systematic effect over time was identified.

### Well-being

**Figure 3:**
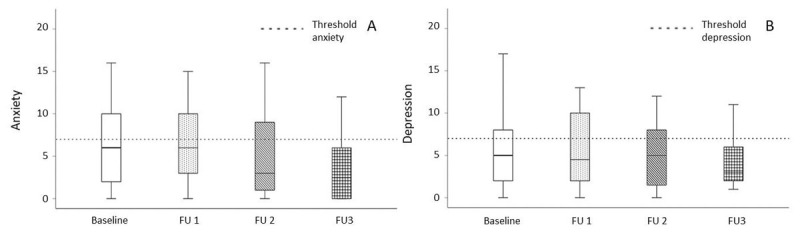
**Anxiety and depression over time**. HADS anxiety (A) and depression (B) scores showed no significant improvement upon follow-up.

Baseline SF-36 scores were below age-related normal values across all domains, with particularly low scores for ‘emotional role limitation’ and ‘energy/fatigue’ (Figure 2). The mean HADS depression (5.6 ± 3.8, n = 52) and anxiety (6.2 ± 4.6, n = 50) scores were below the threshold for potential or suspected depression or anxiety (i.e., >7), but higher than the mean values in the general population (depression/anxiety: 4.8/4.4 for males and 4.7/5.0 for females) (Figure 3) [30]. In total, 56.6% (30 out of 53 patients) had potential or suspected depression and/or anxiety. Among those with depression scores, 26.9% (14 out of 52 patients) had potential depression (scores 8–10), and 9.6% (5 out of 52 patients) had suspected depression (scores >10). For anxiety, 20.0% (10 out of 50 patients) had potential anxiety (scores 8–10) and another 20.0% (10 out of 50 patients) had suspected anxiety (scores >10).

Observed follow-up scores on SF-36 and HADS varied between subject and over time (Figures 2 and 3), but no systematic effect over time was identified in the mixed model. The LMM did show that gender had a significant effect on the sub-score ‘energy/fatigue’, with women scoring higher (p = 0.023). Again, substantial (unexplained) between-subject variability was captured by the random intercepts on all domains (Supplementary Table 1) [29,30].

## Discussion

This study found that overall scores for daily functioning and patient well-being were lower than those of the general population, based on normative values. The majority of patients had scores indicative of potential or suspected depression and/or anxiety. Although most patients perceive OT as a progressive condition, no significant decrease was observed in daily functioning and patient well-being. There was, however, a significant effect of time with a positive difference between baseline and FU3 in the SF-36 sub-score for ‘physical role limitation’. Women had significantly higher scores on the sub-scores ‘physical role limitation’ and ‘energy/fatigue’, after adjustment for baseline age, gender, and disease duration. Additionally, there was substantial variability between patients that remained after adjustments for baseline age, gender, and disease duration, as well as variability within patients across time points.

Few studies have examined daily functioning in OT patients. Two studies (total n = 26) used the iADL and ADL (composite) score, but in patients undergoing DBS, confounding direct comparisons [13,23]. Studies in essential tremor (ET) using iADL scores found high independence in daily activities, similar to our findings, although long-term follow-up was lacking [31,32,33]. The third OT study (n = 16) developed a questionnaire to measure the effect of OT on functioning and QoL over time, showing no significant changes in ADL, mobility, or social participation after six years of follow-up. This is consistent with our results, except for our observed trend toward decreased walking mobility, likely due to different assessment methods with one question in our study vs nine questions in the literature [22], which is similar to SF-36 physical functioning scale, which also showed no decline at follow-up.

Seven studies have assessed patient well-being in OT, with two including long-term follow-up (ranging from 14 to 56 months) [12,21,23,34,35,36,37]. Consistent with our findings, all report decreased well-being compared to the general population, based on SF-36 and HADS [12,21] and other scales [23,34,35,36,37]. However, patients in the studies using the SF-36 had lower scores and longer disease duration (16.0 years) compared to our study (2.6 years). Although in advanced ET and Parkinson’s Disease (PD),disease stage negatively affect daily life, no studies have specifically assessed the effect of disease stage on QoL over time [12,38,39]. Our data, showing stable QoL over time, suggest that disease duration is unlikely to explain the differences in SF-36 scores between studies, which is also supported by a previous study with over six years of follow-up [22,40]. While HADS depression scores in our study are similar to a previous report, anxiety scores are lower [21]. Additionally, the long-term follow-up study of six years indicated a significant emotional impact of OT, though direct comparisons with our results were not possible [22]. Notably, most patients exceeded the threshold for depression and anxiety, a prevalence higher than in PD, a condition known for its neuropsychiatric effect [41]. Thereby underscoring the need to assess these issues in clinical consultations.

OT is widely recognized as a progressive condition that negatively affects patients’ QoL. While many patients perceive OT as progressive, daily functioning and overall well-being did not significantly deteriorate over time. Possibly due to patients becoming more adept at managing their condition and adapting their lives to better suit their abilities, which could open up opportunities for therapeutic interventions. Variability between patients and fluctuations in symptoms across time points may also explain this stability, though the cause—whether related to test retest reliability, patient factors, or other variables—remains unclear. Additionally, the COVID-19 pandemic affected patient care, social interactions, and general well-being, and data-collection. Contrary to our findings, Vijiaratnam et al. (2018) observed no fluctuations, possibly related to fewer assessment points [22]. Similar fluctuations have been reported in OT patients undergoing DBS [23]. Furthermore, differences in group compositions at each follow-up point may have influenced our results.

Potential exacerbating factors for OT, such as fatigue and stress, have not been thoroughly investigated before, though their influence on tremor, and movement disorders in general, is broadly accepted [42,43,44]. The question about weather’s impact was included at the request of patients representatives. Further exploration is needed on how factors like rain, darkness, or extreme heat may influence mobility, mood and overall well-being. Although weather’s effect hasn’t been noted in Parkinson’s and ET, temperature is known to impact functioning in these disorders [45,46]. This may suggest a shared impact of these factors on QoL across different chronic disorders.

Cognitive impairment in OT patients, including executive dysfunction, visuospatial, and memory deficits, may affect perceived QoL due to its role in daily functioning and emotional well-being [47,48,49]. Cognitive dysfunction might be a side-effect of medication, secondary to mood disorders including depression, and/or part of pathological cerebellar involvement. In ET, similar cognitive impairments have been reported, possibly linked to cerebellar dysfunction, which is also known to be involved in tremor generation [48,50,51,52]. Further research is needed to determine the impact on QoL, and origin, of non-motor symptoms, including cognition, in OT.

Our study has several limitations. First, the OT-questionnaire is not validated and its limited context may introduce reporting bias. However, developed in collaboration with the patient community it provides valuable insights that align with their needs and concerns. Second, inconsistent follow-up resulted in varying sample sizes at each follow-up point, complicating statistical analysis, including correlations. Although we found no statistically significant differences in patient characteristics between groups aside of disease duration, the limited sample size reduces statistical power, meaning true differences may have gone undetected. For more details on the power calculation see Supplementary Data. Third, not all OT-questionnaires and self-report scales were completed on the same date, but with a maximum four-month interval for completion and OT’s slow progression, discrepancies were unlikely. Fourth, we did not include a disease specific measure of severity, such as OT-10 [53] or another quantitative measure such as standing time, limiting comparisons to other studies. Finally, medication use and other factors like comorbidity may have affected reported QoL during follow-up. Due to missing data, multiple variables, and the small sample size, we were unable to account for this.

This study adds to the limited research on daily functioning and well-being in OT, particularly their changes over time. Patients in our cohort reported OT to be progressive, with notable variability in walking mobility and some reporting an increasing influence of weather on symptoms. Consistent with existing literature, OT patients had lower well-being than the general population, with over half exhibiting signs of potential or suspected depression and/or anxiety. These findings highlight the need for increasing attention from treating physicians and ongoing research. Despite these challenges, daily functioning and well-being remained stable, possibly reflecting patients’ growing ability to adapt to their condition. However, fluctuations in mean QoL scores suggest inconsistent improvements. Additionally, the observed high inter-individual variability may reflect differences in how OT affects patients. Further research is needed to identify factors affecting QoL, both positively and negatively, as well as factors influencing individual outcomes, as this was limited in this study. Understanding these factors is crucial for improving patient QoL and better addressing individual needs. Especially for patients in professions requiring prolonged standing, since they likely report lower QoL due to functional limitations imposed by OT.

## Data Accessibility Statement

The data that support the findings of this study are available upon reasonable request from the corresponding author, A.F. van Rootselaar.

## Additional Files

The additional files for this article can be found as follows:

10.5334/tohm.1008.s1 Supplementary Data. Power calculation.

10.5334/tohm.1008.s2 Supplementary Table 1. LMM outcomes.

10.5334/tohm.1008.s3 Supplementary Table 2. GLMM outcomes.

## Financial Disclosures

Authors B.S. and J.H. have none financial disclosures for the previous 12 months to report.

Author W.A. Babeliowsky received a grant from Stichting HET REMMERT ADRIAAN LAAN Fonds paid to the institution.

Author R.B. did not receive funding for the current work, the author received research grants Medtronic, Bial, ZonMw, AMC Foundation, ROMO Foundation, and Stichting ParkinsonFonds, all paid to the institution.

Author A.F. van Rootselaar has not received any funding related to the current research. The author received a research grant from Stichting De Merel, paid to the institution.
