# Supplementary material for: Effect of Orthostatic Tremor on Quality of Life – a Cohort Study
Source: Tremor Other Hyperkinet Mov (N Y). 2025 May 7;15:22. doi: 10.5334/tohm.1008 (PMC12063594; doi:10.5334/tohm.1008)
Supplement: Supplementary Data. — Power calculation. [file tohm-15-1-1008-s1.pdf]

Supplementary Data '*Effect of orthostatic tremor on quality of life – A cohort study*'

Power calculation

A small simulation study was performed to inform on the power of the mixed models to find an effect of time. To stay close to the data, we simulated data for samples sizes 50, 35, 30, and 10 at follow-up 0, 1, 2, and 3 respectively. The outcome was standardized, and the true simulated effect of time was linear and was 0 at baseline and 0.7 at follow-up 3. The mixed model was defined as a random intercept model with a fixed categorical effect of time as in the paper. For such a setting, the analysis had a power of 80% over 1000 replications.
