# Supplementary material for: Effect of Orthostatic Tremor on Quality of Life – a Cohort Study
Source: Tremor Other Hyperkinet Mov (N Y). 2025 May 7;15:22. doi: 10.5334/tohm.1008 (PMC12063594; doi:10.5334/tohm.1008)
Supplement: Supplementary Table 1. — LMM outcomes. [file tohm-15-1-1008-s2.pdf]

Supplementary Table 1. LMM outcomes.

Outcome measure		Effect	Estimate	Standard Error	Df.	t value	p value
Daily functioning	Physical role limitation	Fixed Effects					
		Intercept	28.03	36.66	49.88	0.77	0.44813
		FU1	7.87	6.48	79.65	1.21	0.22876
		FU2	12.74	6.89	81.14	1.85	0.06820
		FU3	27.34	10.65	83.73	2.57	0.01205*
		Gender	26.40	9.27	50.10	2.85	0.00635**
		Age	-0.60	0.53	51.20	-1.13	0.26546
		Disease duration	0.32	0.46	57.80	0.71	0.48355
		Random Effects					
		Patient ID (Intercept)	Var= 676.2	SD= 26.00	-	-	-
		Residual	Var= 791.9	SD= 28.14	-	-	-
	Physical functioning	Fixed Effects					
		Intercept	85.291	25.29	45.06	3.37	0.00154*
		FU1	-1.69	3.85	68.44	-0.44	0.66142
		FU2	-2.31	4.13	69.41	-0.56	0.57773
		FU3	-2.09	6.43	69.91	-0.33	0.74618
		Gender	6.56	6.40	46.05	1.02	0.31099
		Age	-0.50	0.37	48.29	-1.36	0.18158
		Disease duration	-0.28	0.31	49.96	-0.91	0.36535
		Random Effects					
		Patient ID (Intercept)	Var= 361.3	SD= 19.01	-	-	-
		Residual	Var= 255.5	SD= 15.99	-	-	-
	Social functioning	Fixed Effects					
		Intercept	41.93	26.25	46.14	1.60	0.117
		FU1	-2.31	4.34	77.45	-0.53	0.595
		FU2	4.58	4.67	78.90	0.98	0.330
		FU3	-3.87	7.26	81.75	-0.53	0.596
		Gender	9.60	6.66	47.30	1.44	0.156
		Age	0.07	0.38	47.29	0.17	0.862
		Disease duration	0.21	0.33	55.37	0.63	0.530
		Random Effects					
		Patient ID (Intercept)	Var= 375.3	SD= 19.37	-	-	-
		Residual	Var= 365.6	SD= 19.12	-	-	-
	iADL	Fixed Effects					

	Intercept	-0.04	2.56	48.21	-0.02	0.987
	FU1	0.27	0.41	81.87	0.65	0.515
	FU2	0.37	0.44	83.77	0.85	0.398
	FU3	0.59	0.69	86.48	0.84	0.402
	Gender	-0.46	0.65	48.37	-0.71	0.482
	Age	0.04	0.04	49.37	1.05	0.298
	Disease duration	-0.01	0.03	57.26	-0.42	0.674
	Random Effects					
	Patient ID (Intercept)	Var= 3.63	SD= 1.90	-	-	-
	Residual	Var= 3.35	SD= 1.83	-	-	-
ADL	Fixed Effects					
	Intercept	6.035	0.31	42.31	19.48	<2e-16***
	FU1	0.092	0.07	78.25	1.29	0.202
	FU2	-0.096	0.07	81.32	-1.29	0.199
	FU3	0.079	0.12	89.81	0.68	0.496
	Gender	0.053	0.08	40.77	0.68	0.501
	Age	-0.00	0.00	44.80	-0.81	0.424
	Disease duration	0.00	0.00	50.40	0.52	0.606
	Random Effects					
	Patient ID (Intercept)	Var= 0.03	SD= 0.17	-	-	-
	Residual	Var= 0.10	SD= 0.31	-	-	-
VAS score symptoms	Fixed Effects					
	Intercept	6.99	2.17	47.68	3.23	0.00227***
	FU1	0.13	0.36	80.90	0.37	0.71372
	FU2	0.13	0.39	84.28	0.35	0.72940
	FU3	0.36	0.60	85.68	0.59	0.55502
	Gender	-0.70	0.55	48.58	-1.27	0.21052
	Age	-0.00	0.03	49.54	-0.01	0.98911
	Disease duration	0.04	0.03	57.00	1.63	0.10899
	Random Effects					
	Patient ID (Intercept)	Var= 2.54	SD= 1.59	-	-	-
	Residual	Var= 2.55	SD= 1.60	-	-	-
VAS score impact	Fixed Effects					
	Intercept	8.33	2.46	49.02	3.38	0.00143**
	FU1	0.55	0.36	78.66	1.54	0.12740
	FU2	0.39	0.39	80.57	1.01	0.31682

		FU3	0.76	0.61	80.93	1.26	0.21079
		Gender	-1.03	0.62	48.63	-1.66	0.10365
		Age	-0.02	0.04	50.13	-0.47	0.64283
		Disease duration	0.03	0.03	55.50	0.92	0.35942
		Random Effects					
		Patient ID (Intercept)	Var= 3.54	SD= 1.88	-	-	-
		Residual	Var= 2.51	SD= 1.59	-	-	-
Patient well-being	Emotional role limitation	Fixed Effects					
		Intercept	129.52	244.24	29.01	0.53	0.600
		FU1	81.67	61.62	67.96	1.33	0.190
		FU2	8.30	64.96	72.58	0.13	0.899
		FU3	13.55	100.17	89.67	0.14	0.893
		Gender	-36.47	61.87	27.68	-0.59	0.560
		Age	0.02	3.57	30.37	0.00	0.997
		Disease duration	-1.64	3.21	38.08	-0.51	0.612
		Random Effects					
		Patient ID (Intercept)	Var= 12953	SD= 113.8	-	-	-
		Residual	Var= 77218	SD= 277.9	-	-	-
	Energy/fatigue	Fixed Effect					
		Intercept	14.44	20.52	46.63	0.70	0.4850
		FU1	1.67	2.92	78.28	0.57	0.5690
		FU2	2.98	3.08	78.44	0.97	0.3370
		FU3	4.98	4.92	80.33	1.01	0.3149
		Gender	12.21	5.19	47.71	2.35	0.0229*
		Age	0.26	0.30	47.78	0.88	0.3837
		Disease duration	0.07	0.25	54.24	0.28	0.7800
		Random Effects					
		Patient ID (Intercept)	Var= 225.9	SD= 16.00	-	-	-
		Residual	Var= 165.0	SD= 12.85	-	-	-
	Emotional well-being	Fixed Effects					
		Intercept	49.02	18.60	47.78	2.64	0.0113*
		FU1	-0.91	2.94	80.15	-0.31	0.7585
		FU2	1.97	3.13	80.49	0.63	0.5303
		FU3	2.32	4.94	83.03	0.47	0.6396
		Gender	5.86	4.71	48.68	1.24	0.2195
		Age	0.13	0.27	49.30	0.47	0.6409

	Disease duration	0.11	0.23	56.32	0.49	0.6243
	Random Effects					
	Patient ID (Intercept)	Var= 194.1	SD= 13.93	-	-	-
	Residual	Var= 167.7	SD= 12.95	-	-	-
Pain	Fixed Effects					
	Intercept	152.70	460.31	23.56	0.33	0.743
	FU1	177.11	124.27	61.37	1.43	0.159
	FU2	4.29	131.98	67.94	0.03	0.974
	FU3	12.71	202.59	92.61	0.06	0.950
	Gender	-92.65	116.35	21.73	-0.80	0.434
	Age	1.49	6.77	24.57	0.22	0.827
	Disease duration	-4.70	6.11	31.43	-0.77	0.448
	Random Effects					
	Patient ID (Intercept)	Var= 27873	SD= 167.0	-	-	-
	Residual	Var= 325909	SD= 570.9	-	-	-
General Heatlh	Fixed Effects					
	Intercept	32.38	21.37	45.20	1.52	0.137
	FU1	0.42	2.55	74.62	0.16	0.870
	FU2	1.89	2.74	75.38	0.69	0.492
	FU3	0.74	4.33	76.20	0.17	0.866
	Gender	6.99	5.40	45.94	1.29	0.202
	Age	0.11	0.31	46.06	0.37	0.715
	Disease duration	0.13	0.34	44.61	0.39	0.700
	Random Effects					
	Patient ID (Intercept)	Var= 300.7	SD= 17.34	-	-	-
	Residual	Var= 125.9	SD= 11.22	-	-	-
Health change	Fixed Effects					
	Intercept	43.21	16.93	44.30	2.55	0.0142*
	FU1	0.05	4.40	85.22	0.01	0.9903
	FU2	-0.91	4.72	91.01	-0.19	0.8477
	FU3	3.18	7.27	106.17	0.44	0.6624
	Gender	5.96	4.27	42.02	1.39	0.1707
	Age	-0.18	0.25	45.87	-0.72	0.4736
	Disease duration	0.085	0.22	55.57	0.38	0.7055
	Random Effects					

	Patient ID (Intercept)	Var= 48.83	SD= 6.99	-	-	-
	Residual	Var= 414.50	SD= 20.36	-	-	-
Anxiety	Fixed Effects					
	Intercept	7.92	4.66	50.64	1.70	0.0952
	FU1	-0.05	0.71	78.69	-0.07	0.9454
	FU2	-0.75	0.73	78.25	-1.03	0.3044
	FU3	-1.78	1.15	78.87	-1.55	0.1252
	Gender	-2.12	1.17	50.81	-1.81	0.0761
	Age	0.02	0.07	51.35	0.26	0.7994
	Disease duration	0.00	0.06	59.10	0.02	0.9837
	Random Effects					
	Patient ID (Intercept)	Var= 12.66	SD= 3.56	-	-	-
	Residual	Var= 8.96	SD= 2.99	-	-	-
Depression	Fixed Effects					
	Intercept	9.83	3.91	47.78	2.51	0.0154*
	FU1	-0.25	0.63	79.76	-0.40	0.6941
	FU2	-0.26	0.65	80.93	-0.40	0.6889
	FU3	-0.53	1.04	83.49	-0.51	0.6098
	Gender	-1.61	0.99	48.11	-1.62	0.1110
	Age	-0.02	0.06	49.07	-0.41	0.6838
	Disease duration	-0.04	0.05	56.86	-0.79	0.4339
	Random Effects					
	Patient ID (Intercept)	Var= 8.46	SD= 2.91	-	-	-
	Residual	Var= 7.50	SD= 2.74	-	-	-

P value: *= 0.05, **= 0.01, ***= 0.001
