# Supplementary material for: Effect of Orthostatic Tremor on Quality of Life – a Cohort Study
Source: Tremor Other Hyperkinet Mov (N Y). 2025 May 7;15:22. doi: 10.5334/tohm.1008 (PMC12063594; doi:10.5334/tohm.1008)
Supplement: Supplementary Table 2. — GLMM outcomes. [file tohm-15-1-1008-s3.pdf]

Supplementary Table 2. GLMM outcomes.

Outcome measure		Effect	Estimate	Standard Error	z value	p value
Daily functioning	Satisfactory walking mobility	Fixed Effects				
		Intercept	8.79	5.52	1.59	0.112
		FU1	-0.50	0.76	-0.60	0.509
		FU2	-0.77	0.84	-0.92	0.360
		FU3	-1.26	1.39	-0.90	0.367
		Gender	-1.02	1.18	-0.87	0.385
		Age	-0.07	0.07	-0.92	0.355
		Disease duration	-0.03	0.06	-0.59	0.557
		Random Effects				
		Patient ID (Intercept)	Var= 8.85	SD= 2.98	-	-
	Sport(ing) activities	Fixed Effects				
		Intercept	3.61	5.67	0.64	0.525
		FU1	-0.94	0.82	-1.14	0.255
		FU2	-0.01	0.87	-0.01	0.994
		FU3	-0.27	1.69	-0.16	0.872
		Gender	-1.50	1.52	-0.99	0.324
		Age	-0.02	0.08	-0.27	0.789
		Disease duration	0.08	0.07	1.27	0.204
		Random Effects				
		Patient ID (Intercept)	Var= 12.71	SD= 3.67	-	-
	Influenced by fatigue	Fixed Effects				
		Intercept	5.15	2.64	1.95	0.0507
		FU1	0.84	0.68	1.23	0.2180
		FU2	-0.89	0.63	-1.42	0.1553
		FU3	-0.43	1.03	-0.42	0.6753
		Gender	-0.48	0.57	-0.84	0.3991
		Age	-0.04	0.04	-1.16	0.2452
		Disease duration	-0.01	0.03	-0.25	0.8009
		Random Effects				
		Patient ID (Intercept)	Var= 0.68	SD= 0.82	-	-
	Influenced by stress	Fixed Effects				
		Intercept	12.92	6.06	2.13	0.0329*
		FU1	0.30	0.77	0.39	0.7005
		FU2	-0.84	0.81	-1.04	0.2973
		FU3	0.79	1.62	0.49	0.6253
		Gender	-2.26	1.30	-1.74	0.0812

	Age	-0.13	0.08	-1.70	0.0884
	Disease duration	0.11	0.09	1.41	0.1573
	Random Effects				
	Patient ID (Intercept)	Var= 8.36	SD= 2.90	-	-
Influenced by weather	Fixed Effects				
	Intercept	-4.23	7.67	-0.55	0.581
	FU1	0.09	0.95	0.10	0.924
	FU2	1.04	1.05	0.99	0.324
	FU3	1.18	1.63	0.73	0.468
	Gender	-0.30	1.84	-0.17	0.869
	Age	0.00	0.10	0.02	0.987
	Disease duration	-0.12	0.13	-0.90	0.366
	Random Effects				
	Patient ID (Intercept)	Var= 46.63	SD=6.83	-	-
OT progressive	Fixed Effects				
	Intercept	8.90	7.16	1.24	0.214
	FU1	-0.21	1.05	-0.20	0.840
	FU2	0.790	1.23	0.64	0.521
	FU3	1.13	1.86	0.61	0.544
	Gender	-1.56	1.83	-0.85	0.394
	Age	-0.01	0.11	-0.06	0.954
	Disease duration	0.03	0.11	0.32	0.751
	Random Effects				
	Patient ID (Intercept)	Var= 52.99	SD= 7.28	-	-

P value: *= 0.05, **= 0.01, ***= 0.001
